# Updated WHO recommendations on antenatal corticosteroids and tocolytic therapy for improving preterm birth outcomes

**DOI:** 10.1016/S2214-109X(22)00434-X

**Published:** 2022-11-16

**Authors:** Joshua P Vogel, Jennifer Ramson, Gary L Darmstadt, Zahida P Qureshi, Doris Chou, Rajiv Bahl, Olufemi T Oladapo

**Affiliations:** a Maternal, Child and Adolescent Health Program, Burnet Institute, Melbourne, VIC 3004, Australia; b Prematurity Research Center, Department of Pediatrics, School of Medicine, Stanford University, Stanford, CA, USA; c Department of Obstetrics and Gynaecology, University of Nairobi, Nairobi, Kenya; d UNDP/UNFPA/UNICEF/WHO/World Bank Special Programme of Research, Development and Research Training in Human Reproduction, Department of Sexual and Reproductive Health and Research, WHO, Geneva, Switzerland; e Department of Maternal, Newborn, Child and Adolescent Health and Ageing, WHO, Geneva, Switzerland

In November, 2022, WHO published updated recommendations for antenatal corticosteroids and tocolytic therapy^1^, ^2^ in response to new evidence about these interventions that superseded the 2015 recommendations about the same interventions.^3^

The benefits and possible harms of antenatal corticosteroids were questioned after the publication of the Antenatal Corticosteroids Trial (ACT) in 2015.^4^ ACT—a cluster-randomised, implementation trial in six low-income and middle-income countries—showed that a multifaceted intervention to increase the use of antenatal corticosteroids did not have any benefits, and led to additional maternal and neonatal harms. This finding from ACT resulted in WHO publishing recommendations in 2015 that defined clear criteria about when antenatal corticosteroids should be used. Primarily, they should be used only when gestational age can be accurately assessed, preterm birth is considered imminent, there is no clinical evidence of maternal infection, adequate childbirth care is available, and the preterm neonate can receive adequate care for complications if needed (eg, resuscitation, thermal care, feeding support, infection treatment, and safe oxygen use).

In the 2022 updated recommendations, the guideline panel considered evidence from 27 efficacy trials, including the WHO ACTION-I trial, which was done in 29 adequately equipped hospitals in five low-resource countries between 2017 and 2019 and incorporated the criteria previously recommended.^5^, ^6^ ACTION-I showed reduced neonatal mortality and respiratory-associated morbidities, with no increase in harms.^6^ This combined evidence showed that the antenatal corticosteroid treatment criteria from WHO can optimise the benefits of this treatment while avoiding the harms observed in ACT. Thus, the guideline panel reiterated these criteria in the updated recommendations, and further consolidated them by specifying Kangaroo Mother Care as a crucial component of neonate care, and that neonate respiratory support should include continuous positive airway pressure as required.

The gestational age threshold for antenatal corticosteroid use remains clinically important. At less than 34 weeks of gestation, there is compelling evidence of reduced perinatal and neonatal mortality and respiratory-related morbidity.^5^ However, after 34 weeks of gestation, there is no difference in mortality, a modest reduction in respiratory morbidity, and increased neonatal hypoglycaemia.^5^ Therefore, until robust evidence that shows an improved balance between benefit and risk becomes available, particularly from low-resource countries, the guideline panel considered that restricting antenatal corticosteroid use to neonates with a gestational age of less than 34 weeks was a reasonable safeguard for preventing unsafe antenatal corticosteroid use, especially when considered in the context of the challenges to accurately estimating gestational age in the late third trimester.

Animal and human studies indicate that there is a relationship between time from antenatal corticosteroid administration to birth and preterm neonate outcomes, although which interval offers the best perinatal outcomes is not clear.^7^, ^8^ Although 1–7 days between treatment and birth seems beneficial, there could be some benefit within the first 24 h of administration, and that benefit might continue after 7 days. However, antenatal corticosteroids should not be administered for individuals who do not have a high likelihood of preterm birth. A 2022 Cochrane review^9^ showed that a repeat course (*vs* no further courses) of antenatal corticosteroids for individuals at ongoing risk of preterm birth more than 7 days after their first course was associated with reduced adverse neonate outcomes, respiratory morbidity, oxygen supplementation, and surfactant use, which could reduce costs. Although there was a small reduction in mean birthweight and an increased risk of being small for gestational age, the guideline panel considered these effects to be clinically less significant than the benefits, and therefore retained the 2015 recommendation of one repeat course of antenatal corticosteroids for eligible women.

In 2015, WHO recommended against the use of tocolytic treatments for spontaneous preterm labour on the basis of no substantive benefit of tocolysis for reducing adverse perinatal or neonatal outcomes.^3^ Evidence was derived from several Cochrane reviews investigating individual classes of tocolytics. Since then, a new Cochrane systematic review and network meta-analysis has been published, summarising evidence from 122 trials (13 697 women) that compared all tocolytic agents and placebo or no tocolytic therapy, and different tocolytic agents against one another.^10^ All classes (ie, betamimetics, calcium channel blockers, magnesium sulfate, oxytocin receptor antagonists, and nitric oxide donors) and their combinations have some effect in delaying preterm birth for at least 48 h, sometimes for 7 days. However, there are clinically important differences across classes for adverse effects (from minor to potentially severe). Overall, three tocolytics—nifedipine, oxytocin receptor antagonists, and nitric oxide donors—had the best benefit–risk profiles. Nifedipine might also reduce rates of respiratory and neurodevelopmental morbidity and low birthweight. Oxytocin receptor antagonists and nitric oxide donors are potential alternatives to nifedipine, as they extend pregnancy and have a good safety profile. However, there is no clear evidence of other perinatal health benefits for oxytocin receptor antagonists or nitric oxide donors; they are not available in many countries and can be costly. Although the network meta-analysis does not suggest a reduction in perinatal mortality with the use of any single tocolytic class, the guideline panel considered the clear benefits of delaying preterm birth for 48 h (and up to 7 days) alongside evidence of extended antenatal corticosteroid administration-to-birth intervals being associated with reduced risk of neonatal mortality. Acknowledging that tocolytic therapy should only be used when there are more benefits than risks for the mother and her baby, the guideline panel specified preconditions for tocolytic therapy to ensure safety.

We declare no competing interests. The WHO guideline development panel that formulated the antenatal corticosteroids and tocolytics recommendations was an independent, international panel of experts convened by WHO. The panel was co-chaired by GLD and ZPQ. JPV was project manager, and RB and OTO were project coordinators for WHO ACTION-I trial. JPV, RB, and OTO did not take part in the formulation of the recommendations.
